# Hyoid Bone Position in Patients with and without Temporomandibular Joint Osteoarthrosis: A Cone-Beam Computed Tomography and Cephalometric Analysis

**DOI:** 10.1155/2021/4852683

**Published:** 2021-12-11

**Authors:** Xueman Zhou, Xin Xiong, Zhebin Yan, Chuqiao Xiao, Yingcheng Zheng, Jun Wang

**Affiliations:** National Clinical Research Center for Oral Diseases, State Key Laboratory of Oral Diseases, Department of Orthodontics, West China Hospital of Stomatology, Sichuan University, No. 14, Section 3, Ren Min South Road, Chengdu 610041, China

## Abstract

**Objective:**

To assess the differences in hyoid bone position in patients with and without temporomandibular joint osteoarthrosis (TMJOA).

**Methods:**

The present cross-sectional study was conducted in 427 participants whose osseous status was evaluated using cone-beam computed tomography and classified into normal, indeterminate osteoarthrosis (OA), and OA. The hyoid bone position and craniofacial characteristics were evaluated using cephalograms. Patients were divided into the normal group (*N* = 89), indeterminate OA group (*N* = 182), and OA group (*N* = 156). Descriptive statistics, one-way analysis of variance, and age- and sex-based stratified analyses were performed. *P* < 0.05 was considered statistically significant.

**Results:**

The differences in Hy to MP, Hy-RGn, Hy to C3-RGn, C3-RGn, and Go-Hy-Me among the three groups were statistically significant. The differences in the Frankfort-mandibular plane angle, saddle angle, articular angle, gonial angle, ramus height, and posterior facial height were statistically significant. After adjusting age and sex, the Hy-RGn and C3-RGn in the normal group were significantly greater than the OA group. No statistical differences were observed in the hyoid measurements in the stratified analyses in males or subjects less than 18 years old. The differences in Hy to MP, Hy to C3-RGn, and Go-Hy-Me in female patients among the three groups were statistically significant. The differences in Hy to SN, Hy to FH, Hy to PP, Hy to MP, Hy-RGn, Hy-C3, Hy to C3-RGn, Go-Hy-Me, Hy-S, and C3-Hy-S in adults were statistically significant.

**Conclusion:**

The differences in the hyoid bone position, mainly relative to the mandible, were statistically significant in patients with or without TMJOA. The difference pattern varied among different age and sex groups. Clinical evaluation of the hyoid position must consider the age and sex of patients. Longitudinal studies are required to clarify the causal relationship between TMJOA and hyoid bone position.

## 1. Introduction

Temporomandibular joint osteoarthrosis (TMJOA), a vital subtype of temporomandibular disorders (TMDs), is a degenerative joint disease characterized by cartilage degradation and subchondral remodeling [1, 2]. Around 27–38% of the general population had TMD [3]. And 11% of the TMD patients have symptoms of osteoarthritis [4]. As a primary chief complaint of TMJOA patients, pain and TMJ dysfunction could compromise the quality of life of patients, causing a considerable social and economic burden [5, 6]. However, the etiology of TMJOA is not yet completely understood.

The hyoid bone is a horseshoe-shaped bone attached to the mandible, skull, pharynx, and cervical spine by different ligaments and muscular attachments. The hyoid bone moves during respiration, mastication, swallowing, and phonation, which are functions affected by the temporomandibular joint (TMJ) [7, 8]. The TMJ, mandible, and hyoid bone are crucial for the functions of the stomatognathic system. Abnormalities of the hyoid bone can also cause pain in the neck, temporal region, TMJ, and mandible [9].

Several studies reported the relation between TMJOA and craniofacial morphology [10, 11]. Patients with TMJOA exhibited the retrusion and clockwise rotation of the mandible [12]. Although no study reported the relationship between hyoid bone position and TMJOA, a few studies investigated the relationship between hyoid bone position and TMD [13–15]. However, the results of these studies were inconsistent.

Therefore, the present cross-sectional study attempted to analyze the differences in hyoid bone position in patients with or without TMJOA, which might help understand the etiology of TMJOA and the management of TMJOA pain.

## 2. Materials and Methods

### 2.1. Study Population

The present cross-sectional study was conducted on 427 patients visiting the orthodontic department of our hospital between 1 January 2020 and 31 July 2021 after institutional ethics clearance. Written informed consent was obtained from all patients or legal guardians. The personal information of all participants was anonymized. Patients with permanent dentition with clear cephalogram and cone-beam computed tomography (CBCT) images at the first visit to our hospital and with the similar osseous status of the left and right joints were included in the study. Patients with tumor or maxillofacial deformity that could cause joint deformity; those with breathing or swallowing disorders; patients with a history of orthodontic treatment, plastic surgery, or other craniofacial surgeries; those with systematic diseases affecting the orofacial regions; and those with a history of TMJ treatment were excluded from the study.

### 2.2. CBCT Evaluation

CBCT was used to evaluate condylar osseous conditions. CBCT scans were performed with a 256-slice CT scanner (J Morita Mfg. Corp., Kyoto, Japan) using the following parameters: tube voltage, 90 kVp; tube current, 5 mA; exposure time, 17.5 s; voxel size, 0.25 m; slice thickness, 0.25 mm; and field of view, 140 × 100 mm^2^. The condylar images were categorized into the following three groups based on the classification of the osseous diagnosis for TMJ [10, 16]:

#### 2.2.1. Normal

The normal size of the condyle, no deformation, subcortical sclerosis, or articular surface flattening.

#### 2.2.2. Indeterminate for Osteoarthrosis (OA)

The normal size of the condyle with subcortical sclerosis or articular surface flattening; no condylar deformation; and condylar hypoplasia with normal condylar morphology but decreased size in all dimensions.

#### 2.2.3. OA

Deformation caused by erosion, osteophyte, subcortical cyst, or generalized sclerosis and short condyles with decreased condylar height but continual cortical bone.

The osseous diagnosis was made by two independent assessors. Any disagreement about the classification was evaluated decisively by a third specialist.

### 2.3. Cephalometrics

**Figure 1:**
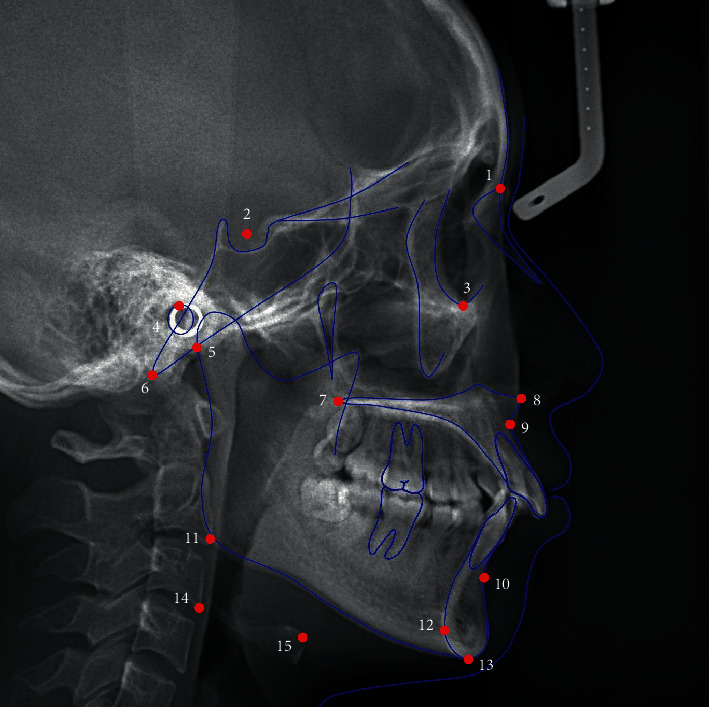
Landmarks used in this study. (1) N: nasion; (2) S: sella; (3) Or: orbitale; (4) P: porion; (5) Ar: articulare; (6) Ba: basion; (7) PNS: posterior nasal spine; (8) ANS: anterior nasal spine; (9) A: A point; (10) B: B point; (11) Go: gonion; (12) RGn: most protrusive point of retrognathion; (13) Me: menton; (14) C3: most anterior and inferior point of the third cervical vertebra; and (15) Hy: most anterior and superior point on the body of the hyoid bone.

All cephalograms were performed as per the standardized technique with natural head position and teeth in centric occlusion. The patients were instructed not to swallow when taking the cephalograms. The digital cephalograms obtained were traced using Uceph software (version 961, Chengdu, China). An experienced orthodontist, blinded to the diagnoses of the patients, performed the cephalogram tracing. The Frankfort horizontal plane was considered the reference plane, and 13 hyoid-related and 18 craniofacial measurements were performed (Figure 1; Table 1) [15, 17]. The intra- and inter-rater reliability of cephalometric tracing was tested, and the intra-class correlation coefficients were >0.8 [17, 18].

### 2.4. Statistical Analysis

Descriptive statistics were presented as mean ± standard deviation. All statistical analyses were performed with the *R* package (http://www.R-project.org, The R Foundation) and Empowerstats (http://www.empowerstats.com, X&Y Solutions, Inc., Boston, MA). An *α* level of 0.05 was considered statistically significant. The diﬀerences in the cephalometric measurements among the groups were evaluated through a one-way analysis of variance (ANOVA) when equal variances were assumed. *P* > 0.05 was considered statistically significant. After the ANOVA test, multiple comparisons between the groups were confirmed by using the S–N–K method. Separate stratified analyses were performed based on sex and age (<18 years vs. ≥18 years).

## 3. Results

### 3.1. Overall Analysis

Of the 427 subjects included in this study, 89 were classified into the normal group, 182 in the indeterminate group, and 156 in the OA group. Subjects in the indeterminate groups were statistically older than the other two groups (*P* < 0.001). The normal group exhibited a higher proportion of males than the other two groups (*P*=0.016; Table 2).

The differences in five hyoid measurements, namely, Hy to MP, Hy-RGn, Hy to C3-RGn, C3-RGn, and Go-Hy-Me, were statistically significant. The differences in craniofacial measurements, namely, Frankfort-mandibular plane angle (FMA), saddle angle, articular angle, gonial angle, ramus height, and posterior facial height among the three groups, were statistically significant (Table 3).

After adjusting age and sex using the generalized additive model, the Hy-RGn and C3-RGn in the normal group were significantly greater than those in the OA group. The differences in ANB, FMA, saddle angle, articular angle, gonial angle, ramus height, posterior facial height, and overbite among the three groups were statistically significant (Table 4).

### 3.2. Stratified Analysis Based on Sex

The female patients in the indeterminate OA group were older than those in the other two groups. The differences in Hy to MP, Hy to C3-RGn, and Go-Hy-Me among the three groups were statistically significant. The differences in FMA, FH-OP, gonial angle, ramus height, posterior cranial base length, and anterior and posterior facial height among the three groups were statistically significant (Table 5).

The male patients in the indeterminate OA group were older than those in the other two groups. No statistical differences were observed in hyoid measurements among the three groups. The differences in FMA, saddle, articular, and interincisal angles among the three groups were statistically significant (Table 6).

### 3.3. Stratified Analysis Based on Age

Patients <18 years of age in the normal group exhibited a greater proportion of males than the other two groups (*P*=0.016). No statistical differences were observed in hyoid measurements. The differences in saddle angle, articular angle, ramus height, posterior cranial base length, posterior facial height, and SNB angle among the three groups were statistically significant (Table 7).

Adults in the indeterminate OA group were significantly older than those in the normal group (*P* < 0.05). The differences in Hy to SN, Hy to FH, Hy to PP, Hy to MP, Hy-RGn, Hy-C3, Hy to C3-RGn, Go-Hy-Me, Hy-S, and C3-Hy-S were statistically significant. The Hy-C3 in the indeterminate group was smaller than that in the normal group. The Go-Hy-Me angle in the indeterminate OA group was greater than that in the other two groups. The differences in the saddle angle, articular angle, ramus height, posterior cranial base length, posterior facial height, and SNB angle were statistically significant. Additionally, the differences among the three groups were statistically significant in ANB, FMA, FH-OP, articular angle, gonial angle, ramus height, and posterior facial height (Table 8).

## 4. Discussion

The present study investigated the hyoid position in patients with or without TMJOA using cephalograms and CBCT. The patients with condylar flattening or subcortical sclerosis were diagnosed with indeterminate OA as these radiological signs were a physiological phenomenon. The patients with indeterminate OA were attempted to be distinguished from those with OA or completely normal condyles. Additionally, patients with inconsistent bilateral osseous status were excluded to prevent cephalometric errors. The differences in hyoid position and Hy to MP and Hy-RGn after adjusting age and sex between the three groups were statistically significant.

Although no study reported the relationship between hyoid bone position and TMJOA, a few studies investigated the relationship between hyoid bone position and TMD. A magnetic resonance imaging study observed that disc displacement was not related to hyoid bone position [14]. Câmara-Souza et al. observed no relationship between TMD and hyoid bone position in 80 dental students [19]. Ekici and Camci reported that the hyoid bone in patients with TMD was located closer to the cranium [15]. The inconsistencies in the findings of these studies may be probably due to inconsistent diagnostic criteria, heterogeneity in sample selection, and methodological differences. Although the relationship between hyoid bone position and TMD is debated, the abnormality of the hyoid bone is often related to cervical painful symptomatology that could be claimed by TMD patients [20, 21]. Nathan et al. detected a release of the hyoid bone away from the floor of the mouth in patients resolved of myofascial pain [22]. These not only support that these structures are anatomically and functionally related but also imply that the position of hyoid bone might be an indicator or a contributing factor of painful TMJOA.

A larger proportion of patients in the OA and indeterminate OA group were female. This finding is concurrent with that of other studies [18, 23]. The patients in the indeterminate OA group were older than the other two groups, with a higher percentage of adults, indicating an age-related change in the condyles [24]. This change could be a normal physiological change [25], resulting from condylar remodeling after mild inflammation or a transition stage to OA [26]. In the OA group, 37.82% were aged less than 18 years, indicating that TMJOA can occur early. Studies have reported the mean age of TMJOA patients as 34 years. This finding is in contrast with that of the present study, where patients with OA were younger. This may be because the patients included in the present study were those undergoing preorthodontic examinations, which consists of most adolescents and young adults. Thus, another population in which TMJOA occurs, namely, the climacteric women aged 40–55 years, was not included.

The present study observed that patients with OA exhibited the largest ANB angle, gonial angle, smaller ramus height, and posterior facial height. The differences in the cephalometric persisted even after adjusting for sex and age, suggesting that patients with OA exhibit clockwise-rotated mandibles with low posterior facial height. This finding is concurrent with that of other studies [10, 27, 28]. Stratified analysis exhibited that ramus height and posterior facial height deficiency were more significant in females, whereas no significant differences were observed in males among the three groups. This may be related to the fluctuations in estrogen. Estrogen has multiple effects on TMJ, such as stimulating bone formation and inhibiting bone resorption [29]. Estrogen levels in women may fluctuate during puberty and near menopause, affecting the stability of the intra-articular environment [30]. On the other hand, androgens are a protective factor in TMD, inhibiting the inflammatory response and reducing pain. Overall, the craniofacial characteristics of the present study population were generally consistent with those of other studies, allowing the generalization of our results.

Ekici and Camci investigated 113 adults, with 55 patients with TMD and 58 healthy volunteers. They observed that adult patients with TMD exhibited hyoid bones closer to the cranium and larger Go-Hy-Me angle [31]. In the present study, adult patients with TMJOA exhibited hyoid bones closer to the cranium and mandible. In contrast, patients with indeterminate OA exhibited a Go-Hy-Me angle larger than the other two groups. The OA group exhibited a slightly larger Go-Hy-Me angle than the normal group. However, this difference was statistically nonsignificant. Possible reasons for this finding might be the older age of the patients in the indeterminate OA group than that in the other two groups or the muscle compensations for the joints demonstrated in the patients with indeterminate OA, which could affect the hyoid bone position. Andrade et al. evaluated the relative position of the hyoid bone concerning the third cervical vertebra. They observed no difference in hyoid bone position between 17 adult patients with TMD and 17 healthy volunteers [31]. The hyoid bone was closer to the third cervical vertebrae in the indeterminate OA group than normal. However, no difference was observed between the OA and the normal group. The distance between the hyoid bone and third cervical vertebrae was related to the upper airway space [9]. Our results revealed that the relative position of the hyoid bone to the third cervical vertebra in patients with indeterminate OA might be more unique. Further studies are required to clarify the characteristics of the hyoid bone position in patients with indeterminate OA.

In the present study, the differences in more parameters among adults and females were statistically significant than between adolescents and males. Adolescents still have growth potential. Therefore, adolescents have more variability in their cephalometric parameters, which may account for the inability to derive statistical differences. For males, indicators such as Hy-RGn did not yield statistical differences due to the relatively small sample size. Further studies with a larger sample size for males may better evaluate the differences in some hyoid indicators. Different patterns of differences in hyoid bone position in different sexes and age groups may be observed. Thus, future studies subdividing the populations are required. In clinical practice, when evaluating the hyoid position, the age and sex of the patient should be considered to obtain an accurate diagnosis.

The main limitation of this study is its cross-sectional design. Therefore, no causal relationship can be built between the hyoid position and OA. Future longitudinal studies are necessary to clarify the causal relationship. Additionally, the hyoid bone and cranium measurements were two-dimensional, and three-dimensional measurements could be used to explore the relationship between the bilateral TMJ and the position and size of the hyoid bone [32].

## 5. Conclusion

Hyoid bone position, mainly relative to the mandible, differs in patients with or without TMJOA. The pattern of differences varies in different age and sex groups. Clinicians should be aware that the patients might have with abnormal position. Clinical evaluation of the hyoid position might be required to consider the age and sex of the patients. Longitudinal studies are required to clarify the causal relationship between TMJOA and hyoid bone position.

## Figures and Tables

**Table 1:** Hyoid and craniofacial measurements from cephalograms in this study.

Measurements	Definition
*Hyoid measurements*	
Hy-Ba (mm)	The distance between Hy point and Basion.
Hy to SN (mm)	The distance between the Hy point and the sella-nasion (SN) plane on a line perpendicular to the SN plane through the Hy point.
Hy to FH (mm)	The distance between the Hy point and the Frankfort horizontal (FH) plane on a line perpendicular to the FH plane through the Hy point.
Hy to PP (mm)	The distance between the Hy point and the palatal plane (PP) on a line perpendicular to the palatal plane through the Hy point.
Hy to MP (mm)	The distance between the Hy point and the mandibular plane (MP) on a line perpendicular to the MP plane through the Hy point. If the Hy point is inferior to the MP plane, the measurement is positive.
Hy-RGn (mm)	The distance between Hy point and RGn point.
Hy-C3 (mm)	The distance between Hy point and C3 point.
Hy to C3-RGn (mm)	The distance between the Hy point and the line formed by the C3 and RGn point on a line perpendicular to the C3-RGn line. If the Hy point is inferior to the C3-RGn line, the measurement is positive.
C3-RGN (mm)	The distance between C3 point and RGn point.
Go-Hy-Me	Angle formed by the Gonion-Hy line and the Hy-Menton line.
Hy-S (mm)	The distance between Hy point and sella.
Hy-C3-S (°)	Angle formed by the Hy-C3 line and the C3-sella line.
C3-Hy-S (°)	Angle formed by the Hy-C3 line and the Hy-sella line.

*Craniofacial measurements*	
SNA (°)	Angle between the SN plane and the nasion-A point line.
SNB (°)	Angle between the SN plane and the nasion-B point line.
ANB (°)	Angle between the nasion-A point line and the nasion-B point line.
Wits (mm)	The distance between vertical lines from A point and B point to the occlusal plane.
FMA (°)	Frankfurt-mandibular plane, formed by the mandibular plane and the FH angle.
FH-OP (°)	Angle formed by the occlusal plane and the FH angle.
Saddle angle (°)	Angle formed by the SN plane and the S-Ar line.
Articular angle (°)	Angle formed by the S-Ar line and the Ar-Go line.
Gonial angle (°)	Angle formed by the Ar-Go line and the mandibular plane.
Interincisal angle (°)	Angle formed the long axis of the upper incisor and low incisor.
Ramus height (mm)	Ar-Go, the distance between articulare and gonion.
Mandibular body length (mm)	Go-Me, the distance between gonion and menton.
Anterior cranial base length (mm)	S-N, the distance between sella and nasion.
Posterior cranial base length (mm)	S-Ar, the distance between sella and articulare.
Anterior facial height (mm)	N-Me, the distance between nasion and menton.
Posterior facial height (mm)	S-Go, the distance between sella and gonion.
Overjet (mm)	The horizontal distance between the upper and lower incisal edge with reference to the occlusal plane.
Overbite (mm)	The vertical overlap between the upper and lower incisal edge.

**Table 2:** Demographic data among the three groups.

	Normal (*N* = 89)	Indeterminate (*N* = 182)	BOA (*N* = 156)	*P*-value
Age (years)	20.32 ± 6.91	24.34 ± 7.69	20.90 ± 7.98	<0.001

Age categorical				
<18 years	35 (39.33%)	39 (21.43%)	59 (37.82%)	<0.001
>=18 years	54 (60.67%)	143 (78.57%)	97 (62.18%)	

Sex				
Female	53 (59.55%)	139 (76.37%)	111 (71.15%)	0.016
Male	36 (40.45%)	43 (23.63%)	45 (28.85%)	

**Table 3:** Differences in hyoid and craniofacial measurements among the three groups.

	Normal (*N* = 89)	Indeterminate (*N* = 182)	BOA (*N* = 156)	*P*-value	
*Hyoid measurements*					
Hy-Ba (mm)	74.01 ± 8.20	73.18 ± 7.06	73.28 ± 8.42	0.698	
Hy to SN (mm)	102.76 ± 9.63	101.21 ± 8.19	102.01 ± 10.07	0.408	
Hy to FH (mm)	84.54 ± 8.39	82.63 ± 7.14	83.51 ± 8.58	0.171	
Hy to PP (mm)	60.03 ± 7.27	58.12 ± 6.19	58.81 ± 7.28	0.098	
Hy to MP (mm)	14.85 ± 5.33	12.05 ± 5.42	13.58 ± 4.95	<0.001	Indeterminate < normal, OA
Hy-RGn (mm)	35.06 ± 5.51	33.69 ± 5.45	32.68 ± 5.28	0.004	OA < normal
Hy-C3 (mm)	33.15 ± 3.99	32.15 ± 3.74	32.36 ± 3.87	0.128	
Hy to C3-RGn (mm)	3.92 ± 9.20	1.12 ± 8.39	1.37 ± 8.58	0.033	OA, indeterminate < normal
C3-RGN (mm)	65.23 ± 7.86	63.60 ± 6.97	62.64 ± 7.02	0.026	OA < normal
Go-Hy-Me	132.47 ± 15.45	140.71 ± 16.61	135.37 ± 15.31	<0.001	Normal, OA < indeterminate
Hy-S (mm)	103.09 ± 9.68	101.54 ± 8.20	102.33 ± 10.09	0.409	
Hy-C3-S (°)	92.48 ± 11.24	91.62 ± 11.33	91.82 ± 11.57	0.840	
C3-Hy-S (°)	94.54 ± 9.56	93.67 ± 7.28	94.23 ± 8.57	0.684	

*Craniofacial measurements*					
SNA (°)	81.79 ± 3.18	82.36 ± 3.69	82.21 ± 3.41	0.451	
SNB (°)	78.88 ± 3.56	79.12 ± 3.80	78.37 ± 4.33	0.217	
ANB (°)	2.92 ± 2.78	3.24 ± 3.11	3.84 ± 3.49	0.065	
Wits (mm)	0.52 ± 3.99	0.23 ± 4.52	0.75 ± 5.34	0.598	
FMA (°)	22.82 ± 5.56	23.31 ± 6.07	25.93 ± 6.39	<0.001	Normal, indeterminate < OA
FH-OP (°)	6.04 ± 4.95	6.57 ± 4.33	7.21 ± 4.63	0.141	
Saddle angle (°)	123.76 ± 4.11	122.90 ± 4.78	121.89 ± 4.58	0.007	OA < normal
Articular angle (°)	150.55 ± 5.57	152.19 ± 6.68	153.67 ± 6.70	0.001	Normal < indeterminate, OA
Gonial angle (°)	117.21 ± 6.80	116.99 ± 7.22	119.30 ± 7.67	0.010	Indeterminate, normal < OA
Interincisal angle (°)	124.68 ± 13.34	124.15 ± 13.73	121.96 ± 13.54	0.213	
Ramus height (mm)	46.09 ± 5.74	46.61 ± 4.83	44.29 ± 5.51	<0.001	OA < normal, indeterminate
Mandibular body length (mm)	69.90 ± 5.69	70.07 ± 5.15	69.15 ± 5.57	0.275	
Anterior cranial base length (mm)	63.48 ± 3.67	63.37 ± 3.36	63.14 ± 3.44	0.730	
Posterior cranial base length (mm)	34.32 ± 3.88	33.94 ± 3.14	33.24 ± 3.98	0.056	
Anterior facial height (mm)	113.78 ± 8.10	114.87 ± 7.68	114.89 ± 8.15	0.512	
Posterior facial height (mm)	77.75 ± 8.24	78.12 ± 6.44	75.42 ± 7.63	0.002	OA < indeterminate
Overjet (mm)	3.89 ± 3.02	4.06 ± 2.79	4.33 ± 3.16	0.501	
Overbite (mm)	2.93 ± 1.67	2.72 ± 1.92	2.36 ± 2.51	0.103	

**Table 4:** Differences in hyoid and craniofacial measurements among the three groups after adjusting age and sex^a^.

	Normal (*N* = 89)	Indeterminate (*N* = 182)	OA (*N* = 156)	*P*-value
*Hyoid measurements*				<0.001
Hy-RGn (mm)	34.86 (33.74, 35.99)	33.80 (33.01, 34.59)	32.66 (31.82, 33.51)	0.002
C3-RGN (mm)	65.10 (63.60, 66.59)	63.56 (62.51, 64.61)	62.76 (61.64, 63.88)	0.016

*Craniofacial measurements*				0.016
ANB (°)	2.88 (2.21, 3.55)	3.24 (2.77, 3.72)	3.85 (3.35, 4.36)	0.017
FMA (°)	22.86 (21.59, 24.13)	23.38 (22.49, 24.27)	25.83 (24.87, 26.78)	<0.001
Saddle angle (°)	123.94 (122.99, 124.90)	122.76 (122.09, 123.43)	121.94 (121.23, 122.66)	0.001
Articular angle (°)	150.71 (149.35, 152.06)	152.01 (151.06, 152.96)	153.79 (152.77, 154.81)	<0.001
Gonial angle (°)	117.07 (115.55, 118.58)	117.27 (116.20, 118.33)	119.07 (117.93, 120.20)	0.021
Ramus height (mm)	45.90 (44.88, 46.93)	46.49 (45.77, 47.21)	44.53 (43.76, 45.30)	0.009
Posterior facial height (mm)	77.23 (75.88, 78.59)	78.13 (77.18, 79.08)	75.70 (74.68, 76.72)	0.023
Overbite	2.88 (2.44, 3.32)	2.74 (2.43, 3.05)	2.36 (2.03, 2.70)	0.049

^a^Data in the table: adjust mean (95% confidence interval); only measurements with statistical differences are illustrated.

**Table 5:** Differences in hyoid and craniofacial measurements among the three groups in female subjects.

	Normal (*N* = 53)	Indeterminate (*N* = 139)	OA (*N* = 111)	*P*-value
Age	22.69 ± 7.25	25.78 ± 7.32	21.85 ± 8.41	<0.001

*Hyoid measurements*				
Hy-Ba (mm)	71.17 ± 7.10	71.28 ± 6.00	70.10 ± 5.80	0.293
Hy to SN (mm)	99.05 ± 7.61	98.81 ± 6.27	97.84 ± 6.62	0.420
Hy to FH (mm)	81.13 ± 6.35	80.67 ± 5.74	80.01 ± 5.96	0.481
Hy to PP (mm)	57.14 ± 5.66	56.58 ± 5.18	55.93 ± 5.06	0.355
Hy to MP (mm)	13.24 ± 4.78	11.14 ± 5.14	12.51 ± 4.61	0.012
Hy-RGn (mm)	34.28 ± 4.76	33.17 ± 4.78	32.49 ± 5.33	0.098
Hy-C3 (mm)	32.28 ± 3.78	31.39 ± 3.19	31.27 ± 3.26	0.170
Hy to C3-RGn (mm)	3.65 ± 7.54	-0.09 ± 7.34	0.23 ± 6.57	0.004
C3-RGN (mm)	64.41 ± 7.11	62.84 ± 5.92	62.34 ± 6.96	0.162
Go-Hy-Me	136.37 ± 15.29	143.20 ± 16.19	137.91 ± 15.02	0.005
Hy-S (mm)	99.34 ± 7.56	99.13 ± 6.30	98.14 ± 6.62	0.407
Hy-C3-S (°)	89.78 ± 11.72	90.83 ± 11.40	89.64 ± 10.39	0.671
C3-Hy-S (°)	92.45 ± 8.50	91.87 ± 6.17	91.92 ± 6.95	0.866

*Craniofacial measurements*				
SNA (°)	81.67 ± 2.90	81.97 ± 3.68	81.91 ± 3.36	0.868
SNB (°)	79.06 ± 3.35	78.84 ± 3.77	78.09 ± 4.14	0.200
ANB (°)	2.61 ± 2.45	3.13 ± 3.05	3.81 ± 3.55	0.055
Wits (mm)	-0.03 ± 3.97	-0.00 ± 4.35	0.21 ± 5.03	0.922
FMA (°)	22.50 ± 5.37	23.85 ± 6.11	26.36 ± 6.15	<0.001
FH-OP (°)	6.16 ± 4.97	6.73 ± 4.34	7.88 ± 4.37	0.039
Saddle angle (°)	123.71 ± 3.82	123.14 ± 4.74	122.48 ± 4.26	0.217
Articular angle (°)	151.06 ± 5.89	152.55 ± 6.81	153.25 ± 6.87	0.148
Gonial angle (°)	116.53 ± 6.12	117.22 ± 7.42	119.95 ± 7.74	0.004
Interincisal angle (°)	124.32 ± 13.97	124.42 ± 14.08	123.25 ± 14.33	0.793
Ramus height (mm)	45.13 ± 4.93	45.85 ± 4.61	42.87 ± 4.65	<0.001
Mandibular body length (mm)	69.29 ± 5.39	69.46 ± 4.61	68.09 ± 5.46	0.089
Anterior cranial base length (mm)	62.75 ± 3.56	62.64 ± 3.04	62.27 ± 3.05	0.553
Posterior cranial base length (mm)	33.26 ± 3.35	33.10 ± 2.55	32.08 ± 3.07	0.009
Anterior facial height (mm)	111.30 ± 6.91	113.95 ± 6.92	112.64 ± 6.68	0.045
Posterior facial height (mm)	75.87 ± 6.72	76.63 ± 5.54	72.84 ± 5.68	<0.001
Overjet (mm)	4.06 ± 2.59	3.86 ± 2.60	4.01 ± 2.98	0.870
Overbite (mm)	2.82 ± 1.50	2.55 ± 1.84	2.29 ± 2.24	0.248

**Table 6:** Differences in hyoid and craniofacial measurements among the three groups in male subjects.

	Normal (*N* = 36)	Indeterminate (*N* = 43)	OA (*N* = 45)	*P*-value
Age	16.83 ± 4.58	19.69 ± 7.06	18.55 ± 6.31	0.123

*Hyoid measurements*				
Hy-Ba (mm)	78.19 ± 8.00	79.33 ± 6.78	81.13 ± 8.80	0.242
Hy to SN (mm)	108.22 ± 9.76	108.96 ± 8.90	112.28 ± 9.80	0.116
Hy to FH (mm)	89.57 ± 8.58	88.98 ± 7.57	92.14 ± 7.98	0.153
Hy to PP (mm)	64.30 ± 7.34	63.12 ± 6.60	65.91 ± 7.09	0.176
Hy to MP (mm)	17.21 ± 5.29	15.00 ± 5.34	16.22 ± 4.83	0.164
Hy-RGn (mm)	36.21 ± 6.34	35.37 ± 6.99	33.16 ± 5.19	0.071
Hy-C3 (mm)	34.42 ± 3.99	34.59 ± 4.34	35.03 ± 3.99	0.782
Hy to C3-RGn (mm)	4.31 ± 11.31	5.02 ± 10.30	4.18 ± 11.83	0.932
C3-RGN (mm)	66.43 ± 8.82	66.05 ± 9.28	63.40 ± 7.20	0.202
Go-Hy-Me	126.73 ± 14.02	132.66 ± 15.51	129.12 ± 14.31	0.196
Hy-S (mm)	108.62 ± 9.91	109.31 ± 8.84	112.68 ± 9.76	0.114
Hy-C3-S (°)	96.46 ± 9.29	94.16 ± 10.85	97.19 ± 12.64	0.422
C3-Hy-S (°)	97.62 ± 10.30	99.50 ± 7.62	99.92 ± 9.54	0.504

*Craniofacial measurements*				
SNA (°)	81.96 ± 3.59	83.62 ± 3.45	82.96 ± 3.43	0.113
SNB (°)	78.60 ± 3.89	80.03 ± 3.81	79.05 ± 4.74	0.298
ANB (°)	3.36 ± 3.18	3.59 ± 3.31	3.91 ± 3.37	0.756
Wits (mm)	1.33 ± 3.93	0.97 ± 5.03	2.09 ± 5.88	0.573
FMA (°)	23.30 ± 5.86	21.57 ± 5.66	24.87 ± 6.91	0.048
FH-OP (°)	5.87 ± 4.98	6.04 ± 4.29	5.57 ± 4.89	0.896
Saddle angle (°)	123.84 ± 4.55	122.09 ± 4.89	120.45 ± 5.05	0.009
Articular angle (°)	149.79 ± 5.05	151.01 ± 6.17	154.71 ± 6.23	<0.001
Gonial angle (°)	118.23 ± 7.68	116.26 ± 6.54	117.71 ± 7.32	0.440
Interincisal angle (°)	125.22 ± 12.51	123.27 ± 12.64	118.77 ± 10.85	0.046
Ramus height (mm)	47.50 ± 6.57	49.04 ± 4.77	47.80 ± 5.93	0.442
Mandibular body length (mm)	70.80 ± 6.06	72.02 ± 6.27	71.76 ± 5.01	0.620
Anterior cranial base length (mm)	64.56 ± 3.59	65.72 ± 3.31	65.30 ± 3.41	0.323
Posterior cranial base length (mm)	35.88 ± 4.13	36.65 ± 3.35	36.10 ± 4.52	0.677
Anterior facial height (mm)	117.43 ± 8.44	117.83 ± 9.21	120.44 ± 8.86	0.241
Posterior facial height (mm)	80.53 ± 9.50	82.92 ± 6.87	81.80 ± 8.11	0.433
Overjet (mm)	3.64 ± 3.58	4.68 ± 3.29	5.13 ± 3.48	0.153
Overbite (mm)	3.08 ± 1.90	3.26 ± 2.07	2.54 ± 3.10	0.359

**Table 7:** Differences in hyoid and craniofacial measurements among the three groups in subjects aged <18 years.

	Normal (*N* = 35)	Indeterminate (*N* = 39)	OA (*N* = 59)	*P*-value
Age (years)	13.30 ± 1.68	13.76 ± 1.86	13.44 ± 1.71	0.496

Sex				
Female	14 (40.00%)	20 (51.28%)	40 (67.80%)	0.026
Male	21 (60.00%)	19 (48.72%)	19 (32.20%)	

Hyoid measurements				
Hy-Ba (mm)	73.16 ± 8.98	75.77 ± 6.81	71.72 ± 8.81	0.066
Hy to SN (mm)	101.70 ± 10.00	104.43 ± 8.20	100.31 ± 10.27	0.119
Hy to FH (mm)	84.19 ± 8.76	85.06 ± 6.65	82.10 ± 8.73	0.187
Hy to PP (mm)	60.16 ± 7.43	60.09 ± 5.74	57.56 ± 7.53	0.117
Hy to MP (mm)	16.44 ± 4.68	14.85 ± 4.22	14.32 ± 5.08	0.109
Hy-RGn (mm)	34.41 ± 6.29	33.49 ± 5.52	32.96 ± 5.46	0.496
Hy-C3 (mm)	32.42 ± 4.17	32.06 ± 4.79	31.00 ± 3.92	0.240
Hy to C3-RGn (mm)	3.17 ± 10.50	1.85 ± 10.08	0.30 ± 9.55	0.392
C3-RGN (mm)	63.20 ± 9.03	62.20 ± 8.08	61.06 ± 7.26	0.445
Go-Hy-Me	126.17 ± 12.68	131.96 ± 12.49	132.59 ± 15.24	0.079
Hy-S (mm)	102.09 ± 10.11	104.83 ± 8.17	100.67 ± 10.37	0.119
Hy-C3-S (°)	93.83 ± 12.17	96.08 ± 9.68	93.62 ± 11.99	0.547
C3-Hy-S (°)	92.70 ± 9.68	94.79 ± 8.40	91.69 ± 7.50	0.203

Craniofacial measurements				0.573
SNA (°)	81.11 ± 3.39	82.92 ± 4.28	82.32 ± 3.68	0.120
SNB (°)	77.42 ± 3.04	79.69 ± 4.14	78.26 ± 4.38	0.048
ANB (°)	3.69 ± 3.03	3.23 ± 2.98	4.05 ± 3.16	0.432
Wits (mm)	1.13 ± 3.86	0.80 ± 4.60	1.40 ± 4.67	0.808
FMA (°)	24.78 ± 5.76	23.81 ± 6.49	25.79 ± 5.59	0.267
FH-OP (°)	7.37 ± 5.48	5.83 ± 4.61	6.70 ± 4.47	0.381
Saddle angle (°)	124.54 ± 4.88	122.18 ± 4.75	121.82 ± 5.25	0.034
Articular angle (°)	149.67 ± 6.19	150.31 ± 5.97	152.61 ± 6.10	0.048
Gonial angle (°)	119.75 ± 6.53	119.23 ± 7.76	120.14 ± 7.45	0.834
Interincisal angle (°)	123.38 ± 13.30	125.45 ± 14.37	121.98 ± 14.79	0.501
Ramus height (mm)	43.35 ± 5.41	45.28 ± 5.03	42.82 ± 3.92	0.038
Mandibular body length (mm)	67.44 ± 5.25	69.62 ± 5.70	67.79 ± 5.79	0.184
Anterior cranial base length (mm)	62.76 ± 3.58	63.31 ± 3.67	62.86 ± 3.57	0.772
Posterior cranial base length (mm)	33.90 ± 3.76	35.05 ± 3.31	33.03 ± 3.90	0.032
Anterior facial height (mm)	112.39 ± 8.64	113.99 ± 8.63	112.23 ± 7.41	0.545
Posterior facial height (mm)	74.50 ± 7.63	77.61 ± 7.12	73.65 ± 6.81	0.026
Overjet (mm)	4.72 ± 2.99	4.64 ± 2.95	5.02 ± 3.10	0.804
Overbite (mm)	3.14 ± 1.80	2.99 ± 2.04	2.67 ± 2.44	0.556

**Table 8:** Differences in hyoid and craniofacial measurements among the three groups in adults.

	Normal (*N* = 54)	Indeterminate (*N* = 143)	OA (*N* = 97)	*P*-value
Age (years)	24.87 ± 4.88	27.23 ± 5.94	25.44 ± 6.79	0.017

Sex				
Female	39 (72.22%)	119 (83.22%)	71 (73.20%)	0.100
Male	15 (27.78%)	24 (16.78%)	26 (26.80%)	

Hyoid measurements				
Hy-Ba (mm)	74.56 ± 7.70	72.47 ± 6.99	74.23 ± 8.08	0.099
Hy to SN (mm)	103.45 ± 9.41	100.32 ± 7.99	103.04 ± 9.86	0.023
Hy to FH (mm)	84.77 ± 8.22	81.97 ± 7.14	84.37 ± 8.42	0.020
Hy to PP (mm)	59.95 ± 7.23	57.59 ± 6.22	59.57 ± 7.05	0.025
Hy to MP (mm)	13.81 ± 5.52	11.28 ± 5.48	13.13 ± 4.85	0.003
Hy-RGn (mm)	35.49 ± 4.95	33.74 ± 5.45	32.51 ± 5.20	0.004
Hy-C3 (mm)	33.61 ± 3.83	32.17 ± 3.42	33.18 ± 3.61	0.016
Hy to C3-RGn (mm)	4.40 ± 8.32	0.92 ± 7.90	2.02 ± 7.91	0.025
C3-RGN (mm)	66.54 ± 6.77	63.98 ± 6.61	63.61 ± 6.73	0.026
Go-Hy-Me	136.55 ± 15.82	143.10 ± 16.83	137.06 ± 15.18	0.005
Hy-S (mm)	103.75 ± 9.44	100.64 ± 8.00	103.34 ± 9.83	0.024
Hy-C3-S (°)	91.61 ± 10.62	90.40 ± 11.48	90.73 ± 11.22	0.796
C3-Hy-S (°)	95.74 ± 9.38	93.37 ± 6.95	95.77 ± 8.84	0.042

Craniofacial measurements				0.048
SNA (°)	82.23 ± 2.99	82.21 ± 3.51	82.15 ± 3.24	0.986
SNB (°)	79.82 ± 3.59	78.96 ± 3.71	78.43 ± 4.32	0.113
ANB (°)	2.41 ± 2.51	3.24 ± 3.15	3.71 ± 3.69	0.062
Wits (mm)	0.12 ± 4.06	0.07 ± 4.51	0.36 ± 5.70	0.902
FMA (°)	21.55 ± 5.07	23.18 ± 5.96	26.01 ± 6.87	<0.001
FH-OP (°)	5.18 ± 4.41	6.77 ± 4.24	7.52 ± 4.72	0.009
Saddle angle (°)	123.25 ± 3.48	123.09 ± 4.79	121.93 ± 4.15	0.084
Articular angle (°)	151.12 ± 5.11	152.70 ± 6.79	154.31 ± 7.00	0.015
Gonial angle (°)	115.57 ± 6.51	116.38 ± 6.97	118.80 ± 7.79	0.010
Interincisal angle (°)	125.53 ± 13.42	123.80 ± 13.57	121.95 ± 12.80	0.267
Ramus height (mm)	47.87 ± 5.26	46.97 ± 4.73	45.19 ± 6.13	0.006
Mandibular body length (mm)	71.49 ± 5.43	70.19 ± 5.00	69.97 ± 5.30	0.199
Anterior cranial base length (mm)	63.95 ± 3.68	63.39 ± 3.29	63.32 ± 3.36	0.512
Posterior cranial base length (mm)	34.59 ± 3.97	33.64 ± 3.03	33.37 ± 4.03	0.121
Anterior facial height (mm)	114.69 ± 7.69	115.11 ± 7.41	116.50 ± 8.20	0.273
Posterior facial height (mm)	79.86 ± 7.99	78.26 ± 6.26	76.50 ± 7.92	0.019
Overjet (mm)	3.36 ± 2.94	3.90 ± 2.73	3.91 ± 3.14	0.464
Overbite (mm)	2.79 ± 1.58	2.64 ± 1.88	2.18 ± 2.55	0.138

## Data Availability

The data used to support the findings of this study are available from the corresponding author upon reasonable request.
